# Poisoning Mechanism Map for Metal Hydride Hydrogen Storage Materials

**DOI:** 10.1002/advs.202408522

**Published:** 2024-09-20

**Authors:** Jiapeng Bi, Panpan Zhou, Wei Jiang, Huaqin Kou, Tao Tang, Yajie Zhang, Yang Liu, Qianwen Zhou, Yunxi Yao, Yuan Zhang, Mao Yang, Lixin Chen, Xuezhang Xiao

**Affiliations:** ^1^ State Key Laboratory of Silicon and Advanced Semiconductor Materials School of Materials Science and Engineering Zhejiang University Hangzhou Zhejiang 310058 China; ^2^ Key Laboratory of Hydrogen Storage and Transportation Technology of Zhejiang Province Hangzhou 310027 China; ^3^ Institute of Materials China Academy of Engineering Physics Mianyang Sichuan 621907 China; ^4^ College of Materials Science and Engineering Hohai University Changzhou 213200 China

**Keywords:** hydrogen storage materials, hydrogenation kinetics, impurity gas, poisoning mechanisms, rate controlling step

## Abstract

The effective utilization of hydrogen storage materials (HSMs) is hindered by impurity gas poisoning, posing a significant challenge for large‐scale applications. This study elucidates the poisoning mechanisms of various impurities gases (CO, CO_2_, O_2_, Ar, He, CH_4_, N_2_) on ZrCo, Pd, U and LaNi_5_. Impurities gases are categorized into active and inactive types based on their effecting behaviors and mechanisms on the hydrogenation of HSMs. During the hydrogenation process, active impurities chemically poison the hydrogenation reaction by limiting hydrogen absorption at interface, while inactive impurities physically hinder hydrogenation reaction by impeding hydrogen diffusion in hydrogen‐impurity mixed gas. In situ Scanning Tunneling Microscope clarifies these behaviors, and a novel criterion based on hydrogen spontaneous dissociation energy is introduced to explain and predict impurity–substrate interaction characteristics. The novel findings of this work provide a comprehensive framework for designing long‐lived HSMs with poisoning resistance, guiding the development of more resilient hydrogen storage systems.

## Graphical abstract

The poisoning mechanisms of impurity gases on hydrogen storage materials are classified into two basic categories. During hydrogenation, inactive gases cause a minimal blanket effect by physically obstructing H_2_ diffusion in H_2_‐impurity mixed gas, while active gases significantly poison materials by chemically limiting H_2_ absoprtion at interface. This understanding informs targeted strategies for comprehensive anti‐poisoning optimizations.

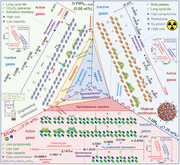

## 1 Introduction

With the continuous depletion of fossil fuels, hydrogen energy is preferred over other sources due to its green, efficient, and sustainable characteristics.^[^ ^1^, ^2^ ^]^ Among mature hydrogen storage techniques, metal hydride‐based solid state hydrogen storage stands out for its high safety, satisfactory volumetric capacity and easy maintenance for large‐scale hydrogen storage and delivery.^[^ ^3^, ^4^ ^]^ However, impurity gases in hydrogen sources inevitably influence the hydrogenation behaviors of HSMs.^[^ ^5^, ^6^, ^7^ ^]^ Understanding the poisoning mechanisms of impurities on HSMs is crucial for enhancing their long‐term service life and ensuring their viability in real‐world engineering applications.

Impurities in H_2_ can decelerate the hydrogenation progress of HSMs to varying degrees.^[^ ^8^, ^9^, ^10^, ^11^, ^12^, ^13^ ^]^ Inert gas He has a minimal blanket effect on hydrogenation kinetics, while CO, CO_2_ and O_2_ tend to significantly impact the hydrogenation kinetics.^[^ ^8^, ^10^, ^11^, ^14^ ^]^ What's worse, the poisoning effect may intensify after multiple de/hydrogenation cycles in mixed gas.^[^ ^6^ ^]^ Current researches on anti‐poisoning modification of HSMs, such as elemental substitution and the development of protective coating, commonly lack theoretical guidance and operate in a black‐box manner.^[^ ^15^, ^16^ ^]^ Despite ongoing efforts, there remains a lack of in‐depth understanding of the magnitude, experimental evidence, and general mechanisms by which various gaseous impurities influence the hydrogen storage performance of HSMs, hindering further anti‐poisoning optimizations.

**Figure 1:**
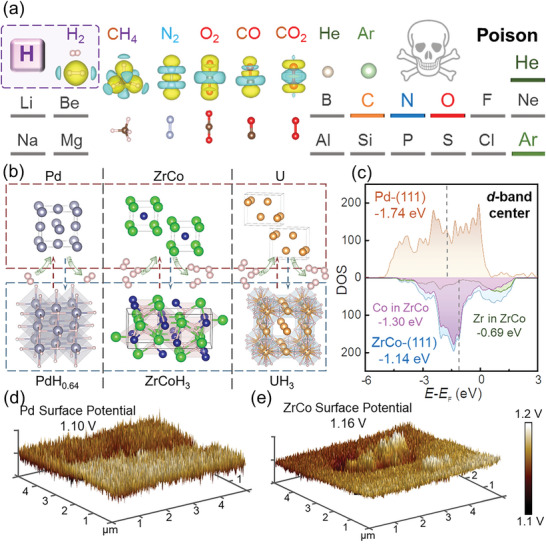
a) Illustration of the impurity gases studied in this work; b) The de/hydrogenation paths for Pd/ZrCo/U; c) Calculated *d*‐band structure for transition metal based ZrCo alloy and Pd; d,e) Mean surface potentials of Pd and ZrCo measured by KPFM.

As a typical scenario, HSMs in the International Thermonuclear Experimental Reactor (ITER) face significant challenges from impurity gas poisoning.^[^ ^17^ ^]^ During ITER operation, high‐temperature hydrogen isotopes including deuterium (D) and tritium (T) plasma ablate structural materials, contaminating hydrogen isotope gases with impurities such as CO_2_, CO, O_2_, N_2_ and CH_4_.^[^ ^15^ ^]^ Additionally, T_2_ decays into ^3^He over time (^3^H→^3^He^+^+β^‐^+*v_e_* +18·6 keV), and Ar is used as a protective atmosphere in the glove box of the tritium containment system.^[^ ^18^ ^]^ These impurities (**Figure** **1**a) must be considered for their potential poisoning effects on ZrCo/Pd/U HSMs, which exhibit the most satisfactory thermodynamic and kinetic properties for ITER as hydrogen isotope mediums (Figure 1b).^[^ ^19^, ^20^, ^21^, ^22^ ^]^

Herein, to investigate general poisoning mechanisms, we systematically explored the effects of seven impurity gases (CO/CO_2_/O_2_/Ar/He/CH_4_/N_2_) on the hydrogenation behaviors of ZrCo/Pd/U. Based on their poisoning characteristics, impurities can be classified as active or inactive species. In detail, active impurities chemically affect hydrogenation by limiting hydrogen absorption at interface, while inactive impurities physically hinder hydrogenation by impeding hydrogen diffusion in hydrogen‐impurity mixed gas. Combining experimental and theoretical calculations, a novel and general H_2_ spontaneous dissociation energy criterion was proposed for the first time to reveal the relationship between the active/inactive nature of impurity gases and the surface electronic structures of HSMs. This insight aids guiding significance for the logical material modification and rational engineering design for practical utilization of hydrogen energy.

## 2 Results and Discussion

Before experimental operation, the fundamental surface properties of U, Pd and ZrCo were first discussed. U, as an actinide metal with active 5*f* electrons and low electronegativity (1.38), is highly susceptible to impurity gases.^[^ ^23^ ^]^ Subsequently, the surface characteristics of transition metals Pd and ZrCo were systemically compared. As depicted in Figure 1c, the calculated *d*‐band centers for Pd and ZrCo surfaces are −1.74 and −1.14 eV, respectively. The absorption energies of a specific impurity gas on substrates containing transition elements linearly correlate with the *d*‐band center of the metal.^[^ ^24^ ^]^ Thus, the lower *d*‐band center of Pd compared to ZrCo suggests its weaker bonding capability to impurity gases. Additionally, the KPFM characterizations (Figure 1d,e) reveal the lower surface potential of Pd (1.10 V) than ZrCo (1.16 V). An elevated surface potential suggests a higher density of surface charges, which enhances the likelihood of impurity gas absorption.^[^ ^25^ ^]^ As a consequence, it can be speculated that the absorption of impurity gases on Pd may not be desirable, resulting in better anti‐poisoning performance compared to ZrCo.

### 2.1 Poisoning of ZrCo

**Figure 2:**
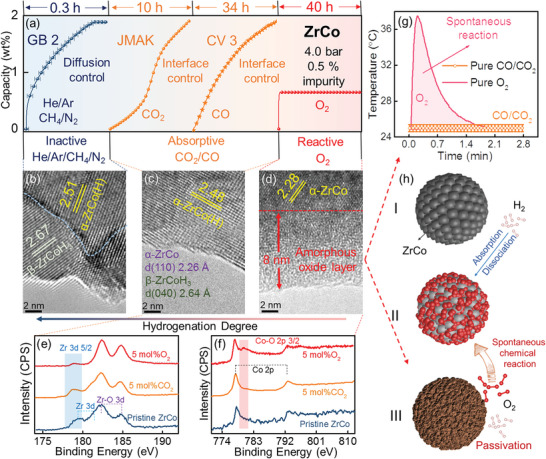
a) Hydrogenation kinetic curves of ZrCo in 4 bar 0.5 mol% CO/CO_2_/O_2_/Ar/He/CH_4_/N_2_ mixed gas; b–d) HRTEM images for ZrCo after being hydrogenated in 1.2 bar 5 mol% CH_4_/CO_2_/O_2_ mixed gas for 0.2 h; e,f) Zr 3d and Co 2p XPS patterns for pristine ZrCo and ZrCo hydrogenated in 1.2 bar 5 mol% CO_2_/O_2_ mixed gas for 0.2 h; g) System temperature variations after introducing pure O_2_/CO_2_/CO into ZrCo; h) Schematic diagram of the poisoning mechanism of reactive active impurity gas O_2_ on the hydrogenation process of ZrCo.

As shown in Figure S2a (Supporting Information), ZrCo achieves its saturated hydrogenation capacity (2.0 wt.%) in pure H_2_ within 1 min. However, when H_2_ is contaminated by CO/CO_2_/O_2_/Ar/He/CH_4_/N_2_ impurities, the H_2_ absorption rate deteriorates to varying degrees, as illustrated in **Figure** **2**a. Among these impurities, He/Ar/N_2_/CH_4_ have minimal impact (Figure 2a‐blue line), enabling ZrCo to achieve its saturated capacity within 0.3 h in 4 bar 0.5 mol% He/Ar/N_2_/CH_4_ mixed gas with almost overlapping kinetic curves. Further kinetic model fitting results indicates that GB 2 model (diffusion‐controlled rate‐controlling step) accurately describes the experimental kinetic curves (Table S1, Supporting Information).^[^ ^26^ ^]^ According to our previous work, the weak physical absorption of “inactive” He/Ar/N_2_/CH_4_ gases that do dot chemically hinder the intrinsic hydrogenation reactivity of ZrCo. The slight decay in hydrogenation kinetics originates from the physical blanket effect. Specially, the selective absorption of H_2_ in hydrogen‐impurity mixed gas separates the inactive gas to form a blanket layer near HSMs, which physically impedes H_2_ diffusion. Worth noting, such processes can be quantitatively analyzed to some extent through mathematical modeling.^[^ ^14^ ^]^

Compared with inactive gases (He/Ar/N_2_/CH_4_), the negative impact of “active” impurities (CO_2_/CO/O_2_) on the hydrogenation kinetics of ZrCo is more significant (Figure 2a). When hydrogenated in 4 bar 0.5 mol% CO_2_/CO mixed gas, ZrCo requires 10 and 34 h to achieve its theoretical capacity, respectively. Unlike inactive gases, kinetic model fitting for the hydrogenation process of ZrCo in H_2_+CO_2_/CO mixed gas^[^ ^26^ ^]^ indicates that the rate‐controlling step is governed by interface‐controlled process. This suggests that the absorption of H_2_ at the interface limits the overall hydrogenation reaction rate. Notably, despite the sluggish hydrogenation kinetics in H_2_+CO_2_/CO mixed gas, capacity saturation can always be reached given enough time. In contrast, as illustrated in Figure 2a‐red line, in 4 bar 0.5 mol% O_2_ mixed gas, ZrCo absorbs 0.65 wt% H_2_ instantly and then completely loses its H_2_ absorption ability.

Based on various poisoning behaviors of impurity gases toward ZrCo, three typical impurities (inactive CH_4_, active CO_2_ and the most toxic O_2_) were chosen to analyze their potential poisoning mechanisms. First, HRTEM analyses were conducted on ZrCo after being hydrogenated in 1.2 bar 5 mol% CH_4_/CO_2_/O_2_ mixed gas for 0.2 h, as shown in Figure 2b–d. The surface hydrogenation degree diminishes noticeably with the transition of impurities from inactive CH_4_ to active CO_2_ and O_2_. For instance, with inactive CH_4_ as impurity gas, the surface of ZrCo absorbs H_2_ to saturation with diffraction stripes (*d* = 2.67 Å) matching well with β‐ZrCoH_3_ *d* (040).^[^ ^27^ ^]^ When active CO_2_ is applied, the surface lattice distance (*d* = 2.48 Å, slightly larger than α‐ZrCo *d*(110) = 2.26 Å) indicates an incompletely hydrogenated α‐ZrCo(H) solid solution, similar to the case of CO shown in Figure S3a (Supporting Information).^[^ ^27^ ^]^ Particularly, O_2_ poisoning results in a distinctive amorphous surface layer (≈8 nm, shown in Figure 2d).

Subsequently, X‐ray photoelectron spectroscopy (XPS) analyses were performed on pristine ZrCo (freshly activated sample) and ZrCo after being hydrogenated in 1.2 bar 5 mol% CH_4_/CO/CO_2_/O_2_ mixed gas for 0.2 h, as displayed in Figure S3b,c (Supporting Information) and Figure 2e,f. For pristine ZrCo, the Zr spectrum exhibits metallic Zr peaks as well as Zr‐O species (inevitably introduced during the sample transfer process^[^ ^28^, ^29^ ^]^) while Co element appears only in tits metallic state. After ZrCo is being poisoned by CH_4_/CO_2_/CO impurity gases, the surface states remain almost identical to pristine ZrCo, indicating CH_4_ (inactive) and CO_2_/CO (active) do not chemically react with ZrCo. Therefore, the poisoning mechanism of active CO_2_/CO impurity gases on ZrCo is the competitive absorption of CO_2_/CO with H_2_ rather than a chemical reaction with ZrCo. Thus, CO_2_ and CO are identified as “absorptive active” gases toward ZrCo.

In contrast, after being exposed to 1.2 bar 5 mol% O_2_ mixed gas for 0.2 h, the surface XPS patterns of ZrCo (Figure 2e,f) exhibit a clear increased oxidation tendency, as could be identified by the almost disappeared metallic Zr component (Zr–O species dominate the surface) and the appearance of a distinct Co–O peak. Combined with HRTEM analysis results, it can be concluded that O_2_ react with ZrCo surface spontaneously to form an amorphous oxide layer. To further confirm the chemical reaction between ZrCo and O_2_, system temperature changes were recorded after introducing pure O_2_/CO_2_/CO gas to ZrCo, as shown in Figure 2g. Notably, when pure O_2_ interacted with ZrCo, the system temperature rose significantly from room temperature (25 °C) to 37 °C, while no obvious temperature fluctuation was observed in pure CO_2_/CO atmosphere. This is the solid evidence for the spontaneous reactivity of O_2_ and thus O_2_ is denoted as an “reactive active” impurity gas toward ZrCo. Therefore, as shown in Figure 2h, one convincing cause for the almost completely cessation hydrogenation for ZrCo in H_2_+O_2_ mixed gas after the initial rapid hydrogenation stage is the O_2_‐induced surface passivation layer.

### 2.2 Poisoning of Pd

**Figure 3:**
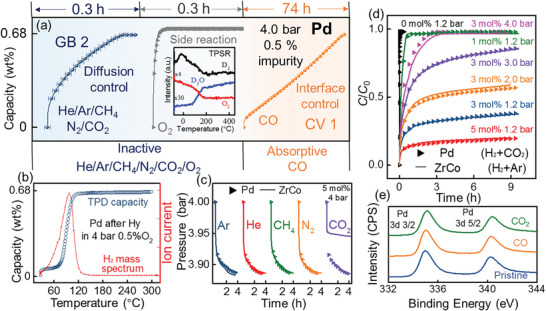
a) Hydrogenation kinetic curves of Pd in 4 bar 0.5 mol% CO/CO_2_/O_2_/Ar/He/CH_4_/N_2_ mixed gas; b) TPD and MS results for Pd after being hydrogenated in 4 bar 0.5 mol% O_2_ mixed gas; c) Hydrogenation kinetic curves for ZrCo and Pd in 4.0 bar 0.5 mol% Ar/He/CH_4_/N_2_/CO_2_ mixed gas; d) Normalized hydrogenation kinetic curves for ZrCo in H_2_+Ar mixed gas and Pd in H_2_+CO_2_ mixed gas with various initial hydrogenation pressures and inactive impurity gas concentrations and e) XPS analysis for pristine Pd and Pd after being poisoned by 4 bar 0.5 mol% CO_2_/CO mixed gas.

Similar to ZrCo, the theoretical capacity (0.68 wt.%) of Pd can also be achieved in pure H_2_ within 1 min (Figure S2b, Supporting Information). As shown in **Figure** **3**a‐blue line, when Pd is hydrogenated in 4 bar 0.5 mol% He/Ar/N_2_/CH_4_ mixed gas, saturated hydrogenation is achieved in only 0.3 h, with kinetic curves almost coinciding with those of ZrCo under same experimental conditions (diffusion‐controlled rate‐controlling step), indicating the inactive nature of Ar/He/CH_4_/N_2_ toward Pd. Surprisingly, the hydrogenation kinetic curve of Pd in 4 bar 0.5 mol% CO_2_ mixed gas is parallel to that in 4 bar 0.5 mol% He/Ar/N_2_/CH_4_ mixed gas (Figure 3a), with no surface reactions identified by XPS (Figure 3e), elucidating CO_2_ is inactive toward Pd surface while being active for ZrCo.

Interestingly, when Pd is reacted in 4 bar 0.5 mol% O_2_ mixed gas (Figure 3a‐grey line), the apparent capacity within 0.3 h (0.79 wt.%, calculated from the pressure drop) slightly exceeds the maximum theoretical capacity of Pd (0.68 wt.%). This suggests that the apparent hydrogenation reaction of Pd is accompanied by a side reaction. As inserted in Figure 3a, TPSR analysis reveals that Pd can catalyze the combination of H_2_ and O_2_ to form H_2_O (catalytic oxidation), contributing to the overcapacity phenomenon of Pd in in H_2_+O_2_ mixed gas.^[^ ^30^ ^]^ After being reacted in 4 bar 0.5 mol% O_2_ mixed gas, Pd was subject to a temperature programmed dehydrogenation (TPD) process (together with MS analysis) to measure the amount and composition of desorbed gas. As shown in Figure 3b, TPD capacity is still around the theoretical capacity (0.68 wt.%), and the main desorbed gas is determined to be H_2_. Namely, in H_2_+O_2_ mixed gas, despite the side reaction during hydrogenation, Pd can still quickly achieve its theoretical hydrogenation capacity. Meanwhile, XRD and XPS analyses for Pd after reacting in H_2_+O_2_ mixed gas exhibit no detectable variations compared with pristine Pd (Figure S4a,b, Supporting Information), and thus O_2_ is also determined as a special inactive gas for Pd.

Blanket effect caused by inactive impurities is actually governed solely by the physical behavior of the gas mixture and thus the hydrogenation kinetic curves in inactive gas contaminated H_2_ atmosphere under same hydrogenation conditions are expected to be coincident regardless of the applied HSMs.^[^ ^14^ ^]^ As demonstrated in Figure 3c, the hydrogenation curves of Pd in 4 bar 5 mol% CO_2_/Ar/He/CH_4_/N_2_ (inactive toward Pd) mixed gas are almost perfect parallel to those of ZrCo in 4 bar 5 mol% Ar/He/CH_4_/N_2_ (inactive toward ZrCo) mixed gas. In contrast, the system pressure drop of ZrCo in 4 bar 5 mol% CO_2_ mixed gas is much slower, further demonstrating that CO_2_ is inactive toward Pd but active toward ZrCo. Afterward, comprehensive hydrogenation tests were conducted (ZrCo in H_2_+Ar and Pd in H_2_+CO_2_ mixed gas) at various impurity gas concentrations (0–5 mol%) and initial hydrogenation pressures (1.2–4.0 bar), as shown in Figure 3d. As expected, the influence rules of inactive gas Ar on hydrogenation kinetics of ZrCo is identical to that of CO_2_ to Pd, further confirming the general applicability of blanket effect.

In contrast to inactive gases, CO shows evident poisoning effect on Pd. As illustrated in Figure 3a‐orange line, when Pd is hydrogenated in 4 bar 0.5 mol% CO mixed gas, it takes 74 h for complete hydrogenation, with CV 1 kinetic model (interface‐controlled rate‐controlling step) determined (Table S1, Supporting Information).^[^ ^26^ ^]^ Notably, the influence of CO on Pd is similar to the influence of CO_2_/CO (absorptive active gas) on ZrCo, where the saturated capacity can be slowly reached with interface‐controlled rate‐controlling step. Furthermore, XPS analysis of Pd after being poisoned by H_2_+CO mixed gas (Figure 3e) exhibits no detectable oxidation or carbonization tendency (only metallic Pd peaks detected), indicating that CO does not chemically react with Pd. Consequently, CO is determined as an absorptive active gas for Pd.

**Figure 4:**
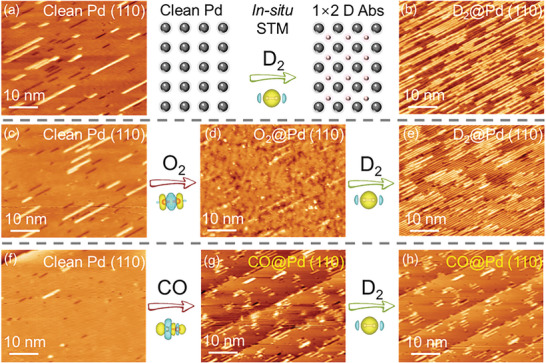
In situ STM pictures of Pd single crystal (110) surface before/after introducing D_2_/O_2_/CO gases.

Subsequently, inactive O_2_ and active CO were chosen as typical impurities to reveal their different influence mechanisms on the hydrogenation reactivity of Pd using In situ STM. As shown **Figure** **4**a,b, introducing hydrogen isotope D_2_ to a clean Pd (110) single crystal surface resulted in a well‐ordered 1 × 2 surface structure, indicating its high reactivity with pure hydrogen. In contrast, O_2_ absorption induced surface roughness and clustering of Pd (Figure 4c,d), which can be completely restored to ordered 1 × 2 surface structure after the subsequent introduction of D_2_ (Figure 4e). This demonstrates that D_2_ absorption is more favorable than O_2_, confirming the inactive nature of O_2_ toward Pd. Notably, CO absorption induced surface reconstruction at defect sites on the Pd surface, forming atomic rows several nanometers long along the (1,−1,0) direction (Figure 4f,g). Specifically, the subsequent introduction of D_2_ into CO‐absorbed Pd (Figure 4h) did not cause any observable change, with typical CO absorption patterns remaining. This confirms that CO preferentially absorbs on the Pd surface over D_2_, severely hindering its hydrogenation reactivity. Thus, CO is an active impurity gas toward Pd, in perfect accordance with the above experimental results.

The inactive/active properties of a specific impurity gas toward ZrCo and Pd are variable. Among the investigated seven impurities (CO/CO_2_/O_2_/Ar/He/CH_4_/N_2_), four impurities are inactive toward ZrCo (Ar/He/CH_4_/N_2_) while six impurities are inactive for Pd (CO_2_/O_2_/Ar/He/CH_4_/N_2_). Moreover, for ZrCo, CO_2_/CO serve as absorptive active gases, whereas O_2_ is a reactive active gas. In comparison, only one active gas is determined for Pd (CO, absorptive active), suggesting its superior anti‐poisoning property.

### 2.3 Absorption Behaviors of CO on ZrCo/Pd Surfaces

**Figure 5:**
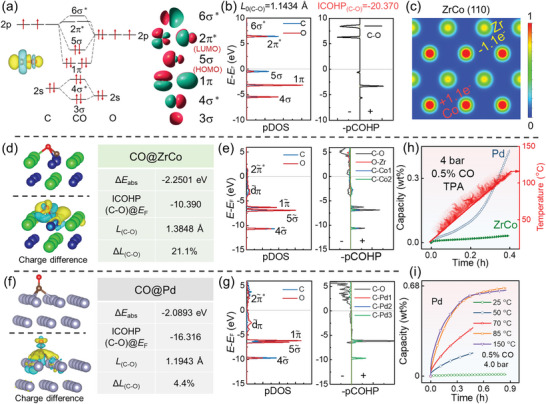
a) Molecular orbitals and b) pDOS and pCOHP curves of gaseous CO; c) Charge analysis result of binary ZrCo (110) surface; Stable absorption configurations and corresponding pDOS and pCOHP curves of d,e) CO@ZrCo and f,g) CO@Pd; h) TPA curves for ZrCo and Pd in 4 bar 0.5 mol% CO mixed gas; i) Isothermal hydrogenation kinetic curves for Pd in 4 bar 0.5 mol% CO mixed gas with various hydrogenation temperatures.

As described above, CO always acts as a strong poisoner both for ZrCo and Pd. The visualized molecular orbitals (MO) of CO are shown in **Figure** **5**a. Due to the higher electronegativity of O (3.44) than C (2.55), the C atom in CO tends to act as an “electron‐accepting center”. According to the model proposed by Blyholder, when CO absorbs on metals, the HOMO 5σ MO of CO hybridizes with the unoccupied orbit of metal electrons, contributing to the main absorption strength.^[^ ^31^ ^]^ As shown in Figure 5a, the charge of 5σ MO is primarily localized at the C‐terminal of CO. Conclusively, C terminal in CO tends to dominate the absorption strength rather than O upon chemical absorption on metals.

Charge analysis of binary ZrCo (110) substrate (Figure 5c) indicates that Co, as the surface negative charge center, is more accessible for “electron‐accepting center” of C in CO. This explains why a stable inclined configuration of CO molecule chemisorbs on ZrCo was optimized (Figure 5d) with Δ*E* of −2.2501 eV, where the carbon terminal is obviously inclined toward the surface Co side. Contrary to binary ZrCo alloy, no obvious charge transfer occurs on single Pd surface, and a stable vertical absorption model (Δ*E* = −2.0893 eV) was obtained with C terminal facing downward, as illustrated in Figure 5f.

For a deeper insight into the absorption behaviors, projected density of states (pDOS) and projected crystal orbital Hamilton population (pCOHP) curves of CO@ZrCo and CO@Pd were further calculated, as shown in Figure 5e,g, respectively. The C‐O bond in CO@ZrCo exhibits obvious anti‐bonding components below Fermi level (*E*_F_), with the calculated ICOHP(C–O) value increasing from −20.370 (gaseous state shown Figure 5b) to −10.390. This indicates that the strong absorption of CO on ZrCo deviates severely from its gaseous chemistry characteristics, corroborated by the alteration in the atomic orbital hybridization pattern (Table S2, Supporting Information) and the apparently elongated C‐O bond (Δ*L* = 21.2%). In contrast, the chemical property of CO after absorption on Pd is similar to that of gaseous CO molecule, as identified by the minimal degree of C─O bond length stretching (4.4%), the higher ICOHP(C─O) value (−16.316), and the unchanged mode of atomic orbital hybridization (Table S2, Supporting Information). Hence, CO absorbed on Pd may be disturbed at elevated temperature.

Afterward, temperature programmed absorption (TPA) experiments were performed for ZrCo/Pd in 4 bar 0.5 mol% CO mixed gas. As shown in Figure 5h, as temperature increases from 25 °C to 120 °C, the amount of H_2_ untaken by ZrCo is negligible while the absorbed H_2_ by Pd exhibits an obvious rise. Therefore, isothermal hydrogenation tests for Pd in 4 bar 0.5 mol% CO mixed gas at various hydrogenation temperatures were conducted. As illustrated in Figure 5i, no detectable amount of H_2_ is absorbed by Pd in 0.9 h at 25 °C, while the hydrogenation speed rises continuously increases with hydrogenation temperature rises to 50, 70, and 85 °C. Specially, at 85 °C, Pd can achieve its saturated capacity within 0.9 h, with almost no additional positive effect on CO tolerance observed at 150 °C. This suggests that the significant poisoning effect of the only active CO on Pd at 25 °C can be effectively eliminated by marginally rising the hydrogenation temperature to 85 °C. Therefore, the Pd‐coated ZrCo sample prepared by electroless plating method (Figure S5, Supporting Information) could essentially eliminate the poisoning effects from all the impurity gases studied in this work.

### 2.4 H_2_ Spontaneous Dissociation Energy Criterion and its Adaption on other HSMs

**Figure 6:**
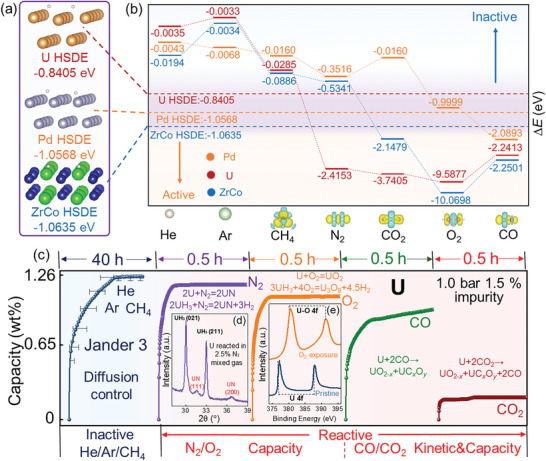
a) Spontaneous dissociation behaviors of H_2_ molecular on U/ZrCo/Pd surface; b) Summary of absorption energies of CO/CO_2_/O_2_/Ar/He/CH_4_/N_2_ impurities and HSDE on U/ZrCo/Pd; c) Poisoning properties and structural characterizations of U by 1 bar 1.5 mol% CO/CO_2_/O_2_/Ar/He/CH_4_/N_2_ impurity gases; d) XRD pattern for U after being hydrogenated in 1 bar 2.5 mol% N_2_ mixed gas; e) XPS analyses for pristine U and U after being exposed to pure O_2_.

The influence of impurity gases on hydrogenation kinetics of substrates may be associated with the intrinsic H_2_ absorption behaviors, as further theoretical calculations were conducted. As shown in **Figure** **6**a, when H_2_ is placed on Pd and ZrCo surface for structure optimization, spontaneous dissociation of H_2_ can occurs with no energy barrier. Specifically, the H‐H distances stretch from 0.7499 Å (molecular H_2_) to 3.0231 and 3.0787 Å, accompanied by H_2_ spontaneous dissociation energy (HSDE) values of −1.0568 and −1.0635 eV, respectively. Since the formation of atomic H is the basic for H diffusion and location in local interstitial sites,^[^ ^26^, ^32^ ^]^ the spontaneous dissociation of H_2_ may play a decisive role in the surface reactivity of HSMs toward H_2_.^[^ ^26^ ^]^

Subsequently, stable absorption states of CO/CO_2_/O_2_/Ar/He/CH_4_/N_2_ on ZrCo/Pd surface (Figure 8; Figure S6, Supporting Information) and the corresponding ∆*E* values are demonstrated in Figure 6b. It is worth noting that impurities with ∆*E* more positive than HSDE have been all identified as inactive gases in our above experiments, which do not hinder the spontaneous dissociation of H_2_ (intrinsic reaction activity of HSMs toward H_2_). Oppositely, impurities with ∆*E* values more negative than HSDE have been proven as active gases that preferentially interact with HSMs surface rather than H_2_. This phenomenon is understandable, as stable system energy is always the inducement of corresponding physical or chemical process.^[^ ^33^ ^]^

Furthermore, this HSDE‐based criterion was applied to predict the poisoning characteristics of impurity gases toward U. As shown in Figure 6a, H_2_ still dissociates spontaneously U surface, with H‐H distance stretching from 0.7499 Å (molecular H_2_) to 3.6355 Å and an HSDE value of −0.8405 eV. Then, the stable absorption behaviors of CO/CO_2_/O_2_/Ar/He/CH_4_/N_2_ on U surface with their corresponding ∆*E* were determined (shown in Figures 6b and 8). Comparing the calculated ∆*E* with HSDE, among the seven investigated impurity gases, only three gases (Ar/He/CH_4_) are speculated to be inactive impurities, while the other four gases (N_2_/CO/CO_2_/O_2_) are identified as active impurities for U.

To confirm the accuracy of the prediction results, the poisoning properties of U by impurity gases were investigated. As shown in Figure S2c (Supporting Information), it takes 4 min for U to achieve saturated hydrogenation capacity (1.26 wt.%) in pure H_2_. When Ar/He/CH_4_ impurities exist in H_2_, typical blanket effect was observed. As demonstrated in Figure 6c, Ar/He/CH_4_ do not affect the hydrogenation capacity of U, with similar influence rule on hydrogenation kinetics (almost overlapping hydrogenation kinetic curves). Meanwhile, similar to the hydrogenation process of ZrCo/Pd in H_2_ contaminated by inactive gases, a diffusion‐controlled rate‐controlling step (Table S1, Supporting Information) for U in H_2_+Ar/He/CH_4_ mixed gas is also identified by kinetic model fitting.^[^ ^26^ ^]^ Moreover, Ar/He/CH_4_ exhibit no detectable chemical reactions with U at room temperature, indicating that Ar/He/CH_4_ exhibit inactive nature toward U.^[^ ^34^ ^]^

On the contrary, N_2_/CO/CO_2_/O_2_ induce a decline in H_2_ absorption capacity of U. After reacting in 1 bar 1.5 mol% N_2_/CO/CO_2_/O_2_ mixed gas for 0.5 h, the achieved capacities decrease from theoretical capacity (1.26 wt.%) to 1.17, 1.07, 0.97, and 0.20 wt.%, respectively. Meanwhile, XRD and XPS results shown in Figure 6d,e) demonstrate that spontaneous chemical reactions occur between N_2_/CO/CO_2_/O_2_ impurity gases and U, forming nitrides, carbides or oxides,^[^ ^34^, ^35^ ^]^ indicating their reactive active nature toward U. Based on the hydrogenation behaviors and structural characterizations, Ar/He/CH_4_ indeed act as inactive impurities for U while N_2_/CO/CO_2_/O_2_ are reactive active impurities. The experimental results align excellently with the predicted poisoning behaviors of U by impurities. This criterion also accurately predicts the poisoning mechanisms of LaNi_5_ (a typical HSM, shown in Figures S7 and S8, Supporting Information) by impurity gases, demonstrating the universality of our proposed HSDE‐based criterion.

### 2.5 Parallel Comparison of Poisoning Behaviors of ZrCo/Pd/U

**Figure 7:**
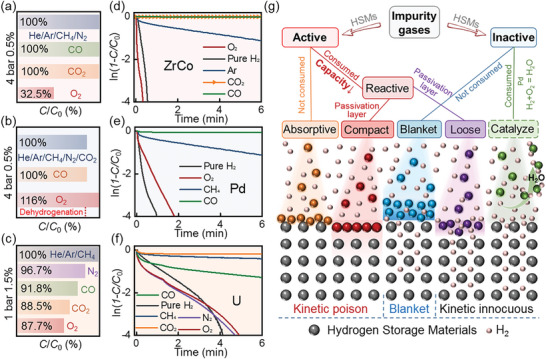
Normalized apparent capacity (*C*/*C*_0_) of a) ZrCo, b) Pd in 4 bar 0.5 mol% mixed gas and c) U in 1 bar 1.5 mol% mixed gas; Hirooka's kinetic model fitting for hydrogenation kinetic curves of d) ZrCo, e) Pd and f) U in mixed gas; g) Influencing mechanisms of various impurity gases on hydrogenation kinetics and capacity of HSMs.

The poisoning effect of impurities on ZrCo/Pd/U HSMs includes both kinetics and capacity aspects. As for capacity, inactive and absorptive active gases do not chemically react with HSMs and thus 100% of theoretical hydrogenation capacity can always be obtained (**Figure** **7**a–c). In comparison, reactive active gases chemically react with HSMs, resulting in a decrease in capacity by consuming substrate to form dead phase.

When it comes to kinetics, Hirooka's model^[^ ^36^ ^]^ was applied to visually compare the initial stage H_2_ absorption behaviors. As shown in Figure 7d–f, inactive gases exhibit non‐negligible negative blanket effect on hydrogenation kinetics (blue line) compared with that in pure H_2_ (black line). The influence of active impurities on hydrogenation kinetics is complex, in which some gases lead to significant kinetic decline (such as CO_2_ on U, CO on ZrCo/Pd and CO_2_ on ZrCo) while others are innocuous on hydrogenation kinetics (such as O_2_ on ZrCo and N_2_/O_2_ on U). More specifically, as illustrated in Figure 7g, absorptive active gases always cause severe poisoning effect on kinetics, yet reactive active impurities may either act as a strong poisoner like absorptive gases or behave innocuous on hydrogenation kinetics. Similar unaffected hydrogenation kinetics for HSMs in H_2_ containing reactive active impurities such as O_2_ are also reported in other researches, indicating that an universal mechanism may underlie these analogical phenomena.^[^ ^10^, ^37^ ^]^ Notably, as a special case, the hydrogenation kinetics of Pd in H_2_+O_2_ (inactive) is also faster than the intrinsic blanket effect caused by other inactive gases such as Ar (Figure 7e). The reason for the above H_2_ absorption processes of HSMs in mixed gas to escape from physical blanket effect deserves further discussion.

As shown in Figure 7g, when impurity gases are consumed either by reacting with HSMs (reactive active impurities) or being catalyzed (e.g., H_2_+O_2_ Pd = H_2_O), no complete impurities‐based blanket layer near HSMs is formed to resist the diffusion of H_2_, accounting for the disappearance of blanket effect. Specially, the influence of reactive active impurities on hydrogenation kinetics may depend on the morphologies of the as‐formed surface passivation layer. A compact passivation layer is able to severely hinder hydrogenation kinetics whereas a loose reacted layer still allows H_2_ to contact fresh HSMs surface for fast H_2_ absorption (innocuous on hydrogenation kinetics).^[^ ^34^, ^37^ ^]^ Overall, the influence mechanisms of impurities on HSMs are summarized in Figure 7g form a kinetic point view, further deepening our understanding on poisoning mechanisms.

**Figure 8:**
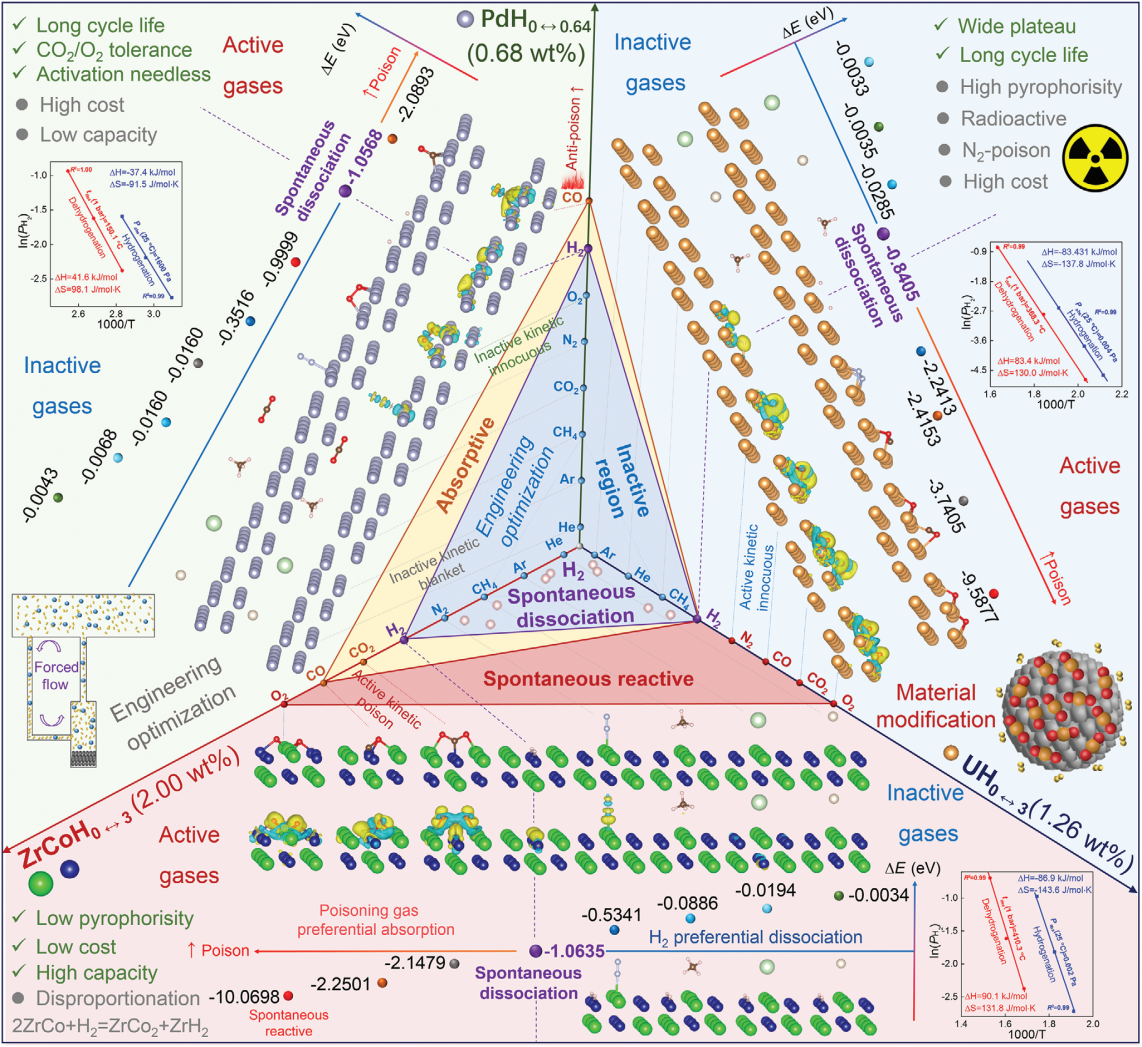
General poisoning mechanism map of CO/CO_2_/O_2_/Ar/He/CH_4_/N_2_ impurities on ZrCo/Pd/U and their corresponding thermodynamic properties for hydrogen storage.

Inspired by the common laws discovered in the influence of gaseous impurities on the hydrogenation absorption behaviors of ZrCo/Pd/U, a comprehensive poisoning mechanism map is summarized, as displayed in **Figure** **8**. The HSDE value reflects the intrinsic hydrogenation reactivity of an HSM. For instance, the specific HSDE values determined for ZrCo, Pd, U are −1.0635, −1.0568 and −0.8405 eV, respectively. This established general HSDE baseline (purple boundary) can successfully distinguish and predict the inactive (Δ*E* > HSDE) and active (Δ*E* < HSDE) natures of impurity gases toward various HSMs. Specifically, the particular active gases for U/ZrCo/Pd (the rest are inactive) are N_2_/CO/CO_2_/O_2_, CO/CO_2_/O_2_ and CO, respectively. A common feature observed is that He/Ar/CH_4_ always act as inactive impurities toward hydrogen storage substrates, while CO invariably serves as an active poisoning gas. Active impurities show significantly more deleterious kinetic impact (interface‐controlled rate‐controlling step) or capacity attenuating effect on the hydrogenation absorption process of HSMs than inactive gases (diffusion‐controlled rate‐controlling step) and thus act as most toxic components in hydrogen storage systems.

## 3 Conclusion

In this study, we introduced a novel H_2_ spontaneous dissociation energy criterion (HSDE) to distinguish and predict the active or inactive nature of impurity gases affecting HSMs. During hydrogenation, active impurities poison HSMs by chemically limiting hydrogen absorption at interface while inactive impurities cause blanket effect by physically impending H_2_ diffusion in hydrogen‐impurity mixed gas. Drawing from the disparate effect mechanisms of active and inactive impurities on HSMs, targeted anti‐poisoning strategies should be customized as appropriate. For instance, to mitigate the negative effects of inactive impurities, engineering optimizations to disturb blanket layer rather than material modifications are favorable approaches. Conversely, to reduce the impact of active impurities, modifying HSMs substrate is highly effective to desensitize HSMs surface toward impurities gases. In summary, our proposed general HSDE‐based criterion provides vital guidance for composition optimization and surface modification for HSMs to improve poisoning tolerance. The aforementioned methods can realize the amelioration of anti‐poisoning properties for HSMs in specific circumstances, shedding light on the rational construction of powerful modification measures for advanced all‐around properties for practical application.

## 4 Experimental Section

### Materials Preparations

ZrCo alloy ingot was obtained via induction levitation melting under Ar protection environment using 99.8% Zr and 99.9% Co (mass %) as raw materials. Subsequently, activation processes of ZrCo alloy and U (99.9%) by de/hydrogenation cycles in pure H_2_ were carried to get activated ZrCo and U for subsequent property measurements. Pd (99.9%) was used directly after purchased (Hebei Enyi Metal Materials) without initial activation. Further characterizations of HSMs used in the experiment are illustrated in Figure S1 (Supporting Information).

### Microstructural Characterizations

In this work, the phase structures of HSMs were determined by X‐ray diffraction (XRD, PANalytical) using Cu K*_α_* radiation. The surface structures were characterized via scanning electron microscope (SEM, Hitachi S 3400‐I) and high‐resolution transmission electron microscopy (HRTEM, Tecnai G2 F20). In addition, the gas composition was identified by mass spectrometry (MS, Hiden QIC‐20) and temperature programmed surface reaction (TPSR). Surface elemental states were determined by X‐ray photoelectron spectroscopy (XPS, ESCALAB 250Xi) using Al K*_α_* radiation. The surface potentials of materials were measured by Kelvin probe force microscope (KPFM, Bruker Dimension Icon). Finally, the absorption behaviors of impurity gases were determined via In situ Scanning Tunneling Microscope (STM) using Pd single crystal (110) surface as substrate. Considering that hydrogen isotopes H_2_ and D_2_ have similar influence rules on hydrogen storage performance, STM experiments used D_2_ instead of H_2_ to simulate real ITER scenarios.

### Theoretical Calculations

ZrCo (110), Pd (111), U (021) and LaNi_5_ (100) slabs were established using Vienna ab initio simulation package program with projected augmented wave method. For the calculation of the interaction of gases toward material surface, a vertical vacuum space of 15 Å was applied with a kinetic energy cutoff of 520 eV. The Perdew–Burke–Ernzerhof generalized‐gradient approximation was selected as the exchange‐correlation functional.^[^ ^38^, ^39^ ^]^ Convergence criteria for total energies and residual forces were set to be 10^–6^ eV and 0.01 eV Å^−1^, respectively. Monkhorst‐Pack grid k‐points of 3 × 2 × 1, 3 × 3 × 1, 2 × 2 × 1 and 3 × 2 × 1 was adopted for the geometric optimization of ZrCo, Pd, U and LaNi_5_ slabs, respectively. Bader charge analysis was performed to determine the magnitude of charge transfer.^[^ ^40^, ^41^ ^]^ In addition, crystal orbital Hamilton population (COHP) analysis was adopted with LOBSTER software to uncover the C‐O bonding form for both gaseous and absorbed CO.^[^ ^42^, ^43^ ^]^ Finally, the projection of density of states and visualization of optimized geometric structures and charge differences were performed using P4VASP and VESTA software.

### Statistical Analysis

For hydrogen storage measurements, a Sieverts‐type apparatus was used. The amount of absorbed/desorbed H_2_ was calculated using the ideal gas equation based on system pressure changes measured by a pressure sensor. In some cases, to compare the hydrogenation kinetic curves of different HSMs with varying theoretical hydrogen storage capacities, an equivalent capacity (*C*/*C*_0_) was defined to normalize the theoretical capacity to 1. Here, *C*_0_ represents the theoretical amount of H_2_ that should be absorbed by unpoisoned HSMs, while *C* is the actual amount of absorbed gas determined from the system pressure drop.

## Conflict of Interest

The authors declare no conflict of interest.

## Author Contributions

J.B. and P.Z. contributed equally to this work. J.B. performed conceptualization, data curation, investigation, methodology, validation, visualization, wrote the original draft, reviewed and edited the final manuscript and acquired Software. P.Z. performed methodology, validation, visualization, wrote the original draft, reviewed and edited the final manuscript and acquired Software. W.J. performed investigation, validation, reviewed and edited the final manuscript. H.K. reviewed and edited the final manuscript. T.T. performed Funding acquisition, acquired resources, Supervision, and reviewed and edited the final manuscript. Y.Z. performed investigation, reviewed and edited the final manuscript. Y.L. performed investigation, methodology, validation, and reviewed and edited the final manuscript. Q.Z. performed data curation, validation, and reviewed and edited the final manuscript. Y.Y. performed funding acquisition, acquired Resources, Supervision, and reviewed and edited the final manuscript. Y.Z. performed methodology, and reviewed and edited the final manuscript. M.Y. performed methodology, and reviewed and edited the final manuscript. L.C. performed funding acquisition, acquired Resources, Supervision, and reviewed and edited the final manuscript. X.X. performed Funding acquisition, Project administration, acquired Resources, Supervision, and reviewed and edited the final manuscript.

## Supporting information



Supporting Information

## Data Availability

The data that support the findings of this study are available from the corresponding author upon reasonable request.
