# Supplementary material for: Poisoning Mechanism Map for Metal Hydride Hydrogen Storage Materials
Source: Adv Sci (Weinh). 2024 Sep 20;11(43):2408522. doi: 10.1002/advs.202408522 (PMC11578344; doi:10.1002/advs.202408522)
Supplement: Supplementary file 1 — Supporting Information [file ADVS-11-2408522-s001.docx]

**Poisoning Mechanism Map for Metal Hydride Hydrogen Storage Materials**

Jiapeng Bi ^a,†^, Panpan Zhou ^a,d,†^, Wei Jiang ^c^, Huaqin Kou ^c^, Tao Tang ^c,^*, Yajie Zhang ^a^, Yang Liu ^a^, Qianwen Zhou ^a^, Yunxi Yao ^c,^*, Yuan Zhang ^c^, Mao Yang ^c^, Lixin Chen ^a,^*, Xuezhang Xiao ^a,b,^*

^a^ *State Key Laboratory of Silicon and Advanced Semiconductor Materials; School of Materials Science and Engineering, Zhejiang University, Hangzhou 310058, Zhejiang, China*

^b^ *Key Laboratory of Hydrogen Storage and Transportation Technology of Zhejiang Province, Hangzhou 310027, China*

^c^ *Institute of Materials, China Academy of Engineering Physics, Mianyang 621907, Sichuan, China*

^d^ *College of Materials Science and Engineering, Hohai University, Changzhou 213200, China*

^*^ Corresponding author. Tel./fax: +8657187951876.

*E-mail address*: xzxiao@zju.edu.cn (X.Z. Xiao); tangtao@caep.cn (T. Tang); yaoyunxi@caep.cn (Y.X. Yao); lxchen@zju.edu.cn (L.X. Chen).

^†^ The authors contributed equally.

*Supplementary Table*

**Table S1.** Kinetic model fitting results for the hydrogenation kinetic curves of ZrCo/Pd/U in mixed gas containing various impurities.


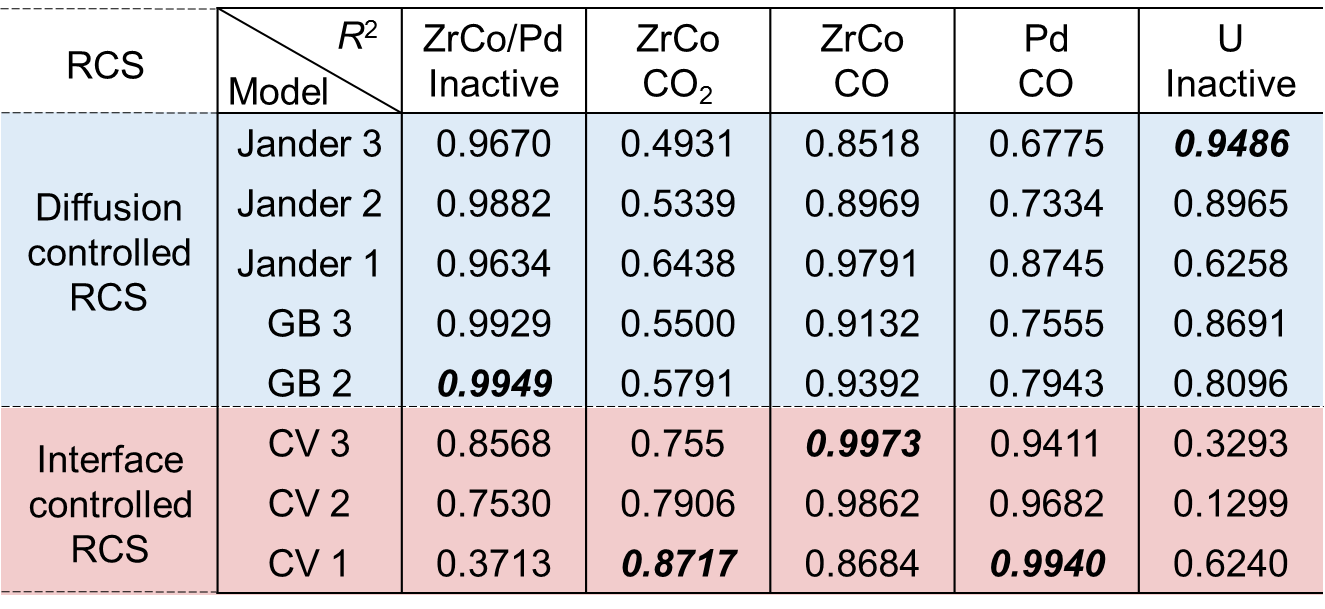


**Table S2.** ICOHP results of gaseous CO, CO absorbed on ZrCo and CO absorbed on Pd.


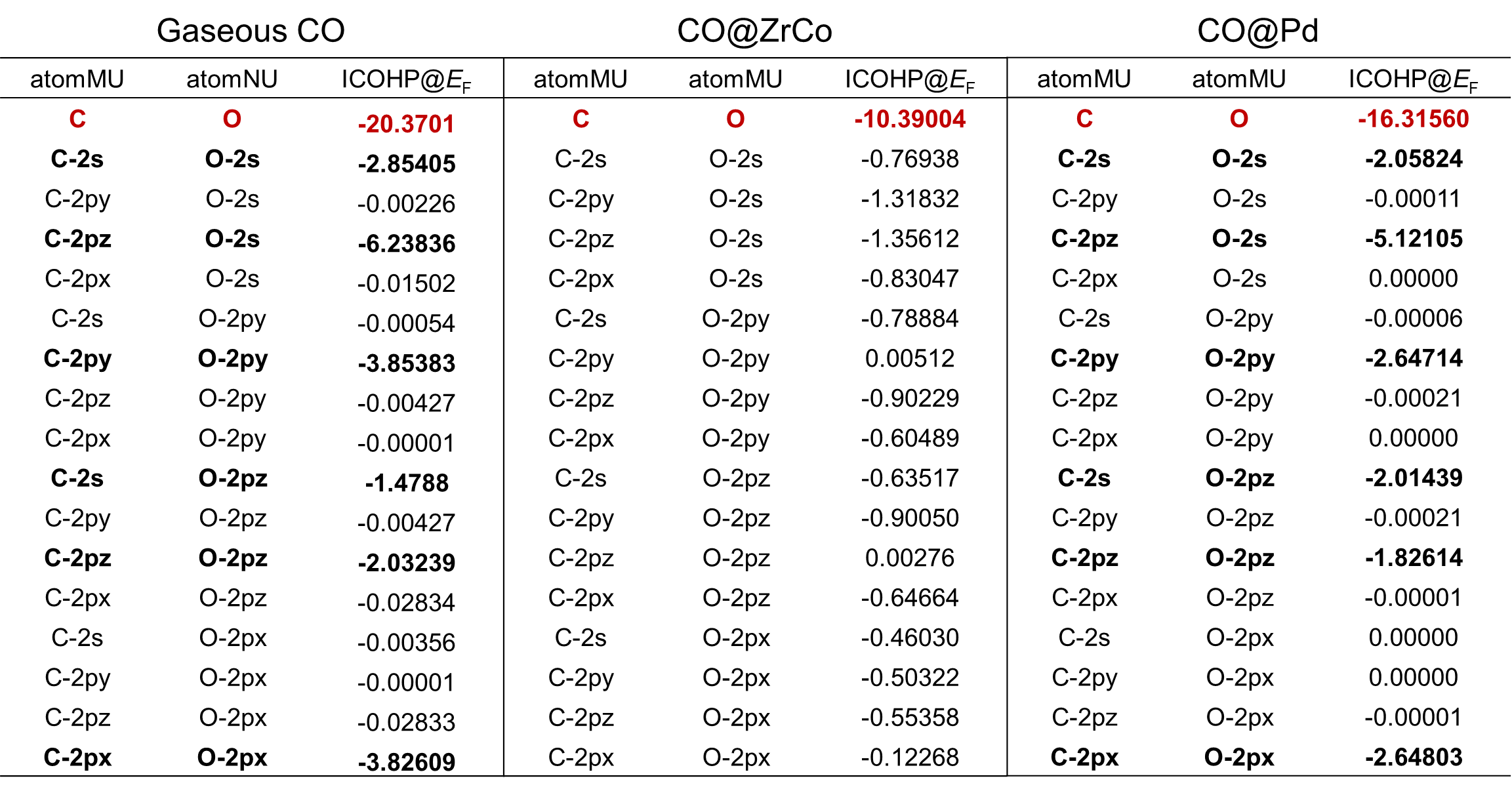


*Supplementary Figures*


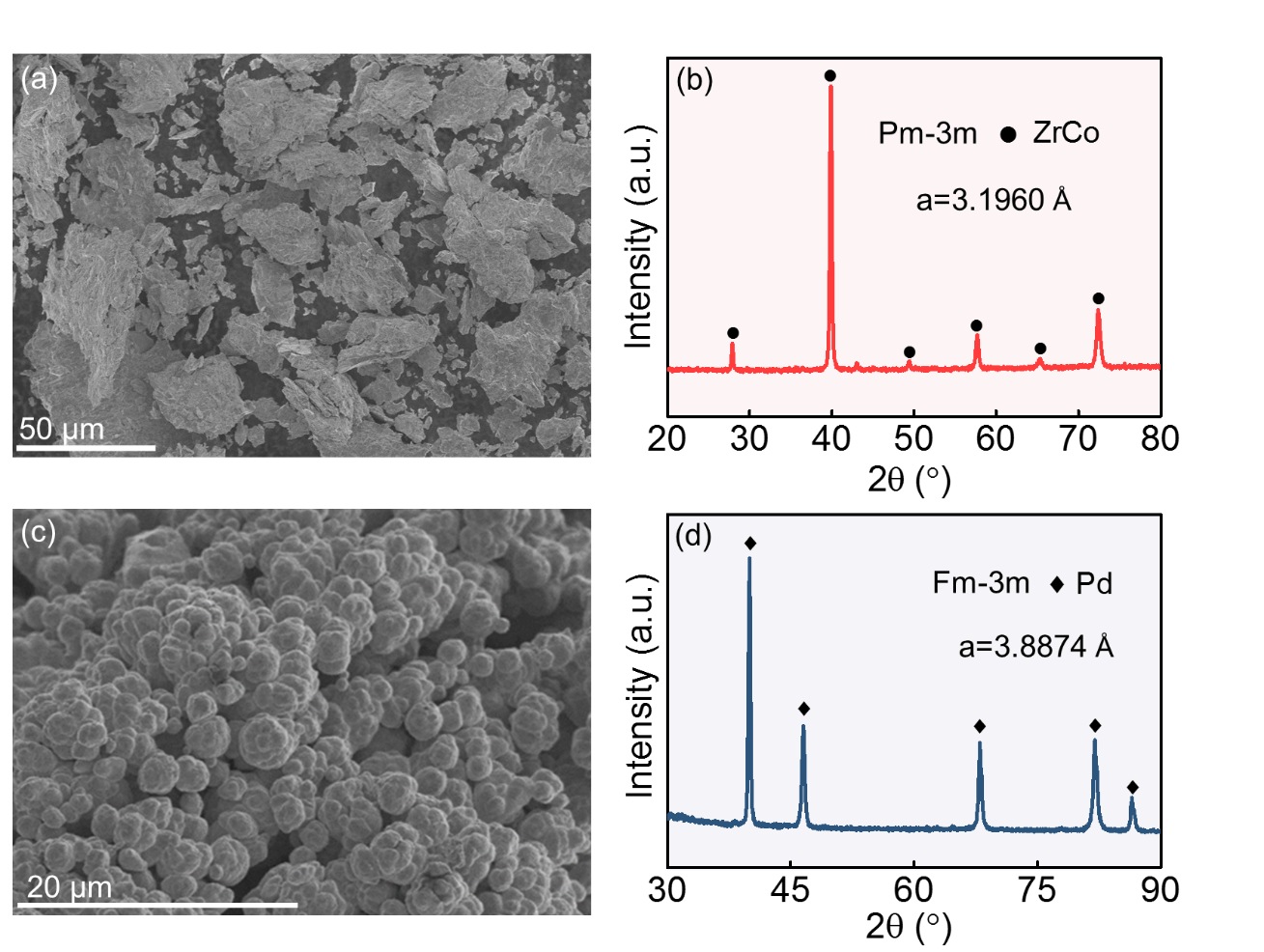


**Fig. S1.** (a,c) SEM images for as-activated ZrCo and Pd used in our experiment and (b,d) their corresponding XRD patterns.


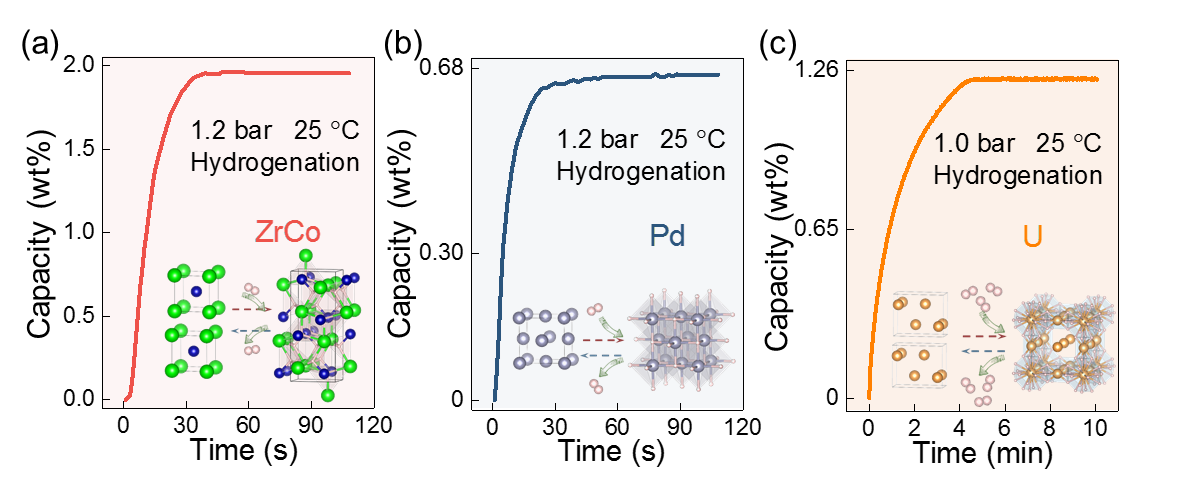


**Fig. S2.** Hydrogenation kinetic curves of (a) ZrCo, (b) Pd and (c) U in pure H_2_.


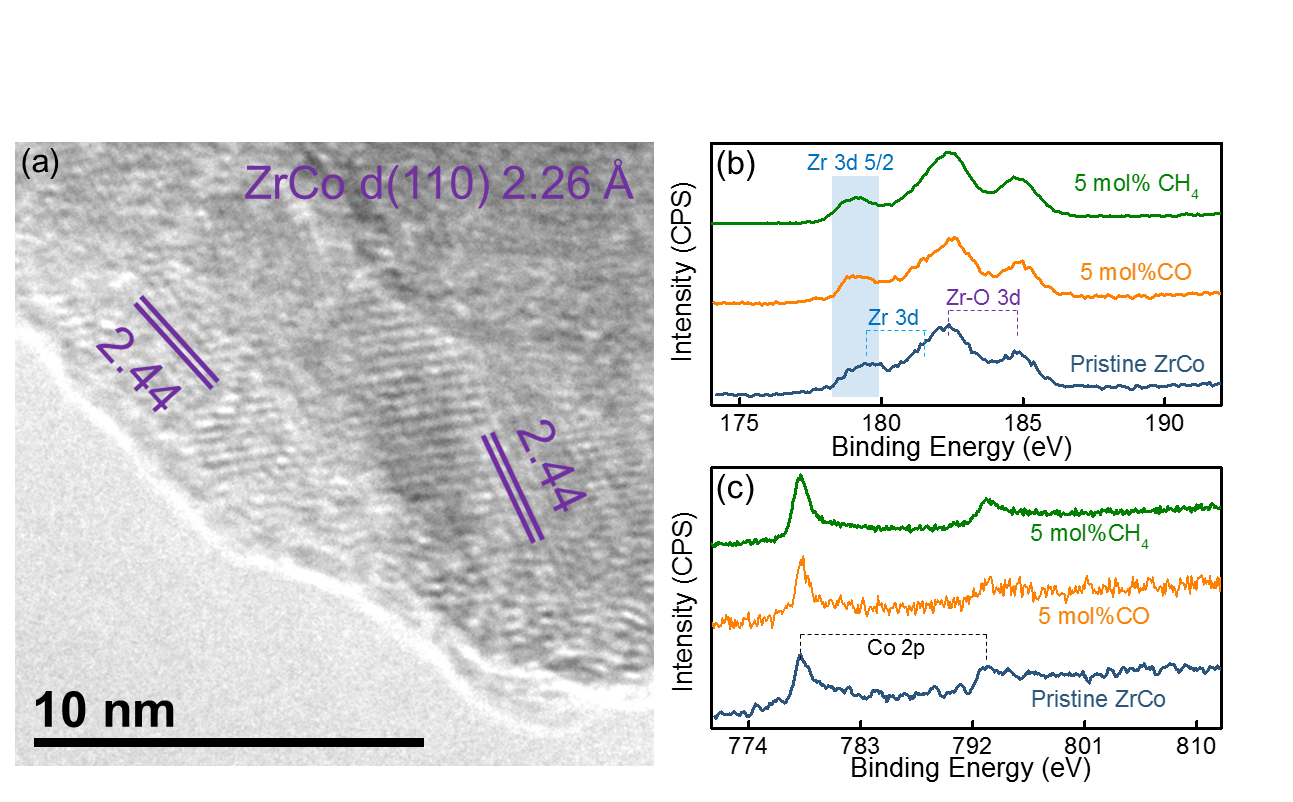


**Fig. S3.** (a) HRTEM analysis for ZrCo after being hydrogenated in 1.2 bar 5 mol% CO mixed gas for 0.2 h; (b-c) Zr 3d and Co 2p XPS patterns for ZrCo after being hydrogenated in 1.2 bar 5 mol% CH_4_/CO mixed gas for 0.2 h.


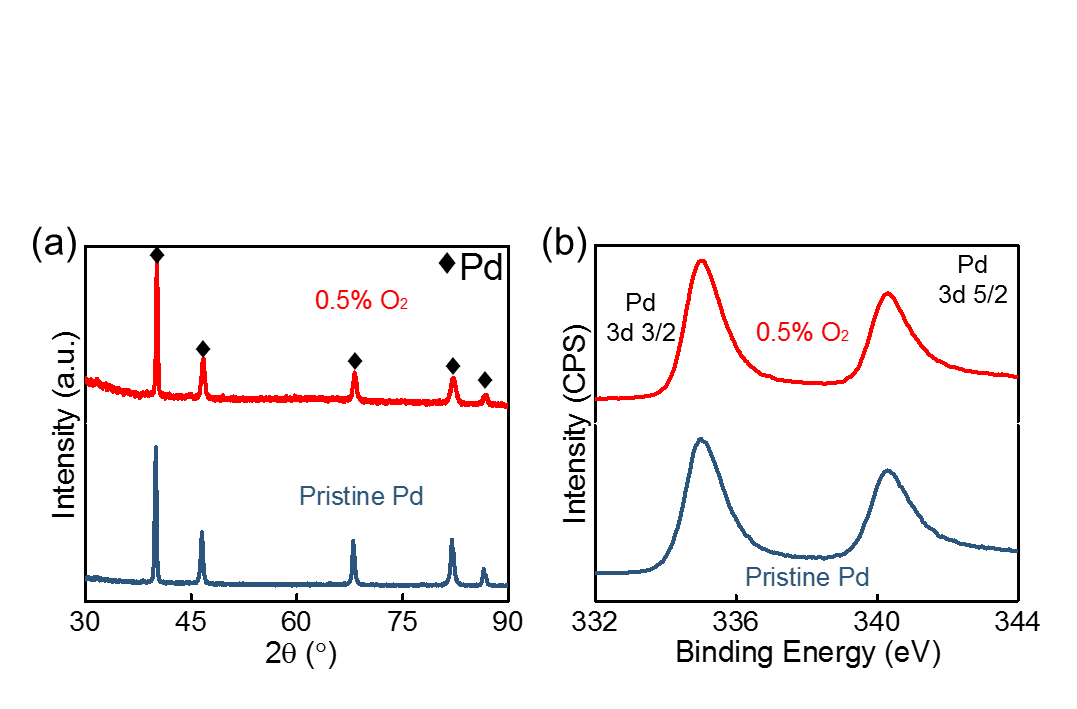


**Fig. S4.** (a) XRD analysis and (b) XPS patterns for pristine Pd and Pd after being reacted in 4 bar 0.5 mol% O_2_ mixed gas.


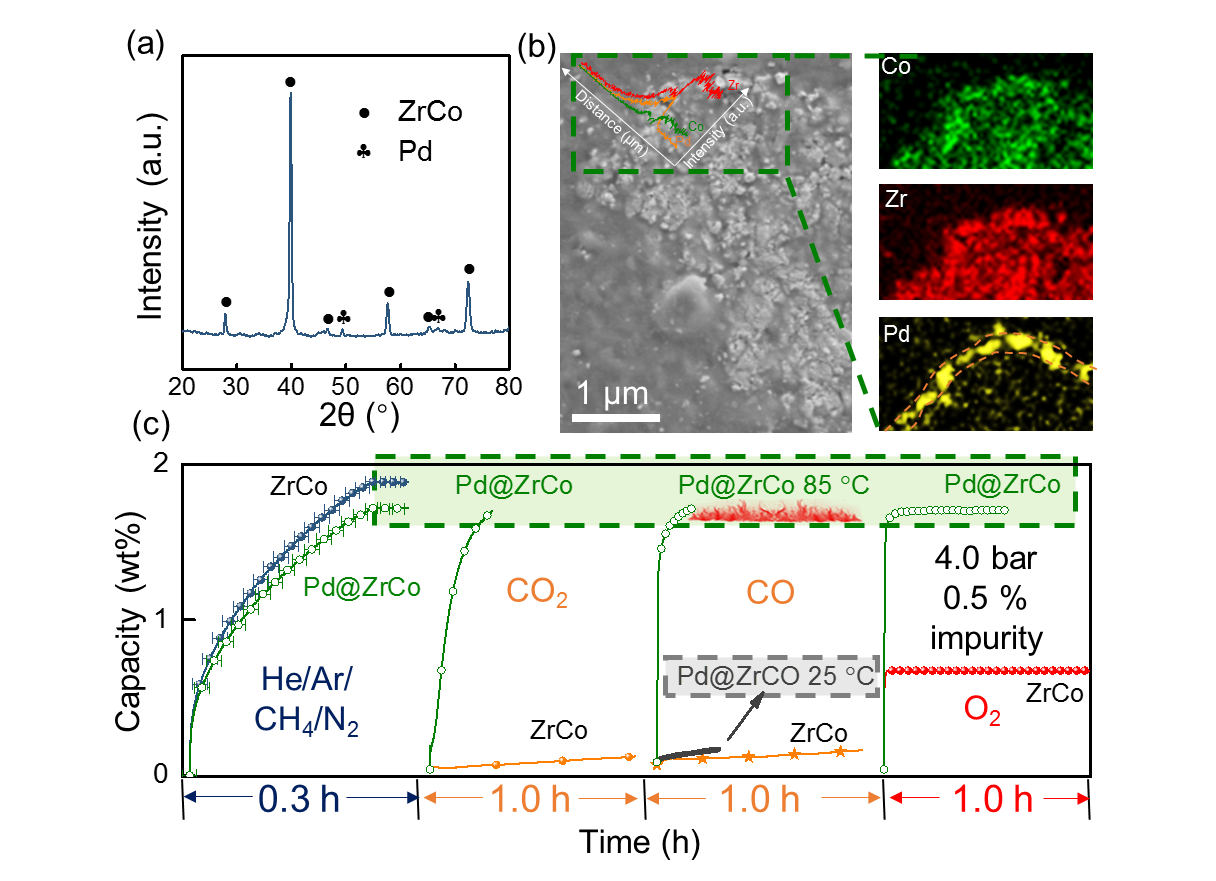


**Fig. S5.** (a) XRD patterns; (b) SEM and EDS results and (c) hydrogenation kinetic curves for pristine ZrCo and Pd@ZrCo samples in H_2_ containing various impurities.

The detailed method for electroless palladium plating is presented in our previous work [S1].


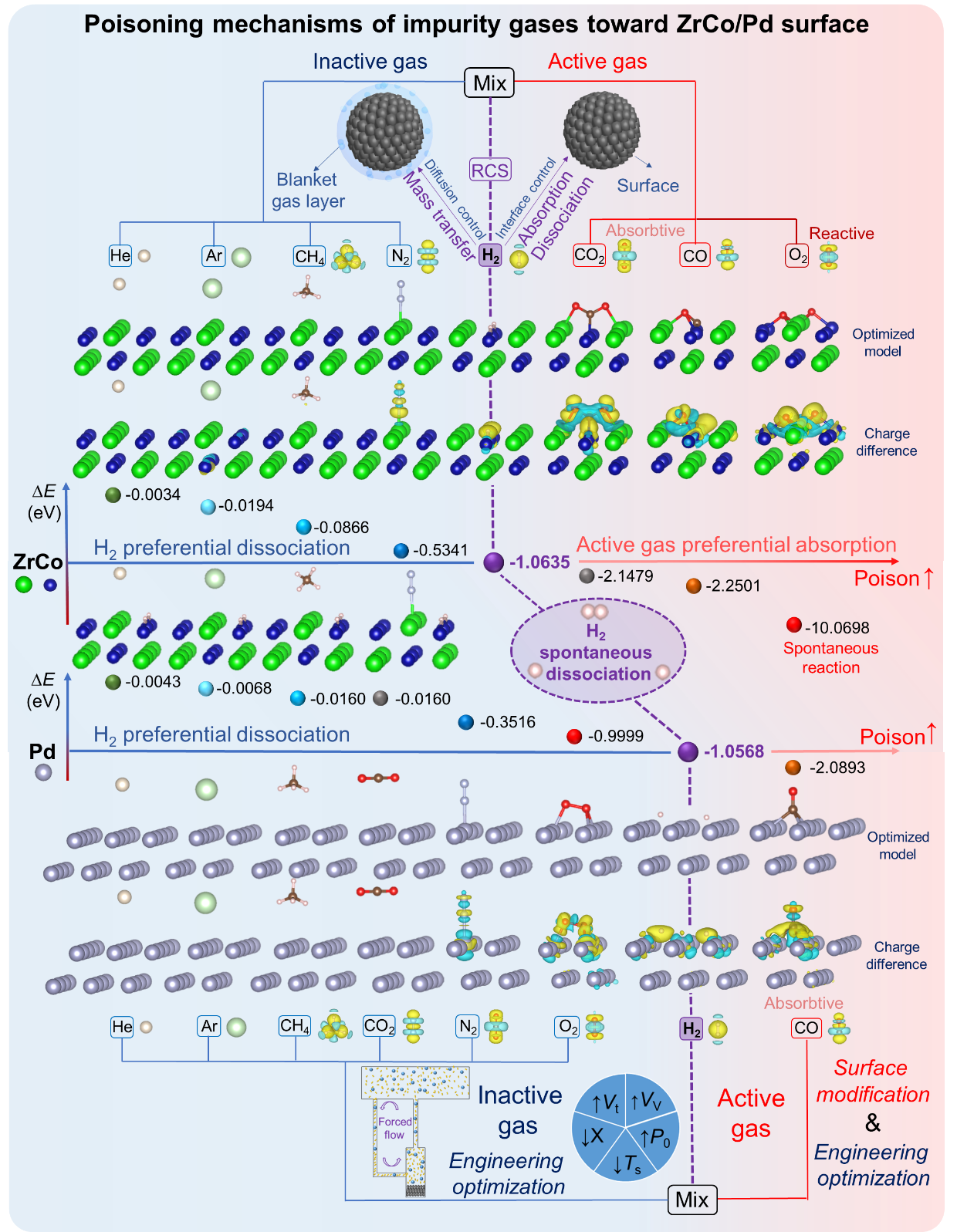


**Fig. S6.** Poisoning mechanism map of CO/CO_2_/O_2_/Ar/He/CH_4_/N_2_ impurities on ZrCo/Pd surface.

To further reveal the adaptation of our proposed H_2_ spontaneous dissociation criterion (HSDE), the spontaneous dissociation behavior of H_2_ and absorption behaviors of He/Ar/CH_4_/N_2_/CO_2_/CO/O_2_ were calculated on LaNi_5_ (100) surface **(Fig. S7)**. The HSDE of H_2_ on LaNi_5_ surface is determined to be -1.5374 eV. According to the proposed rule, four impurities He/Ar/CH_4_/N_2_ (with absorption energies more positive than HSDE) should exhibit inactive nature while CO_2_/CO/O_2_ (with absorption energies more negative than HSDE) are active. This is in well accordance with the experimental results **(Fig. S8)** and reported literatures [S2-3], further confirming the generality of this criterion.


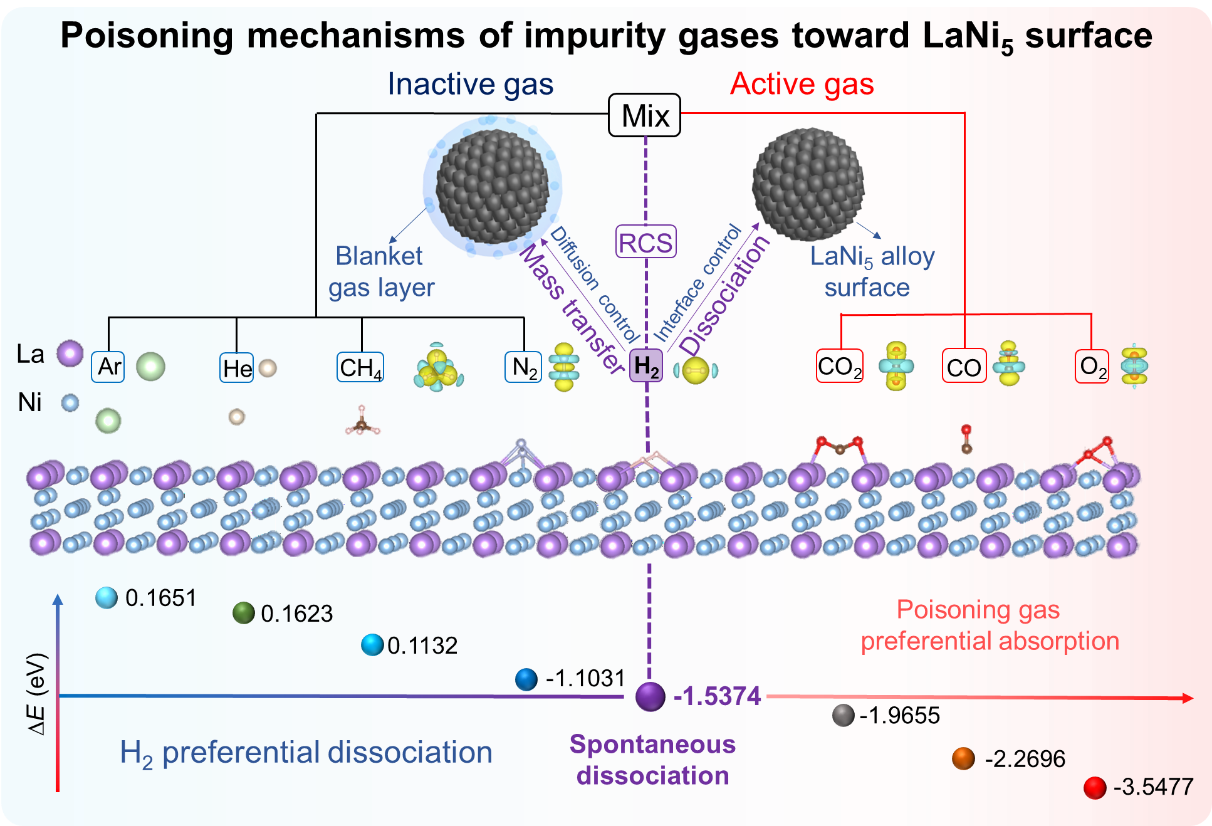


**Fig. S7.** Poisoning mechanism map of CO/CO_2_/O_2_/Ar/He/CH_4_/N_2_ impurities on LaNi_5_ surface.

***
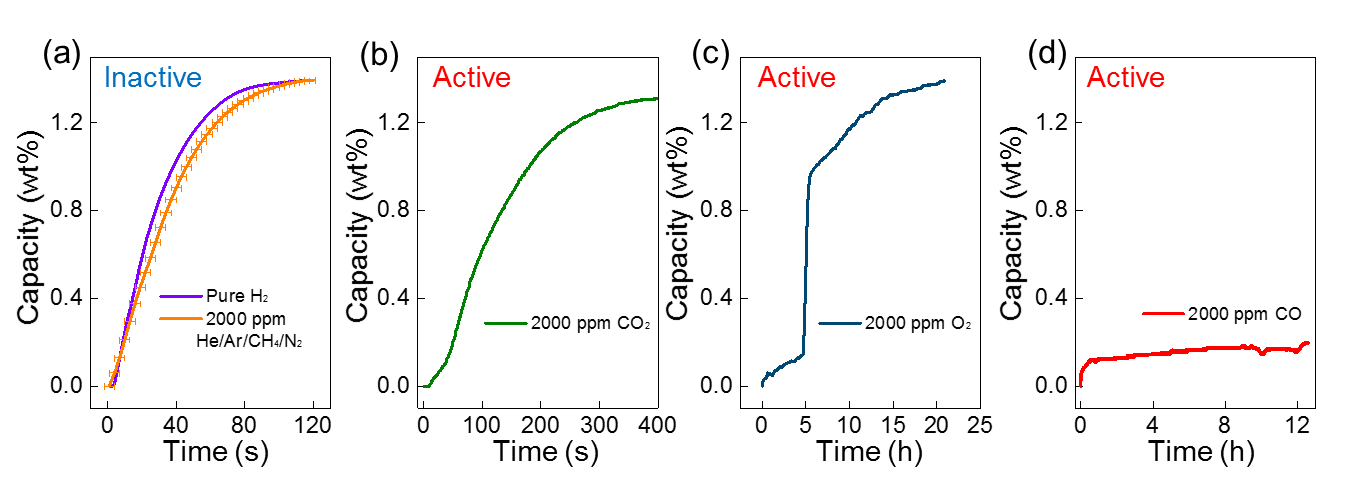
***

**Fig. S8.** Hydrogenation kinetic curves of LaNi_5_ in 10 bar 2000 ppm He/Ar/CH_4_/N_2_ CO/CO_2_/O_2_ mixed gas.

**References**

[S1] Y. Zhang, P. Zhou, X. Xiao, J. Bi, X. Zhang, H. Kou, X. Huang, T. Tang, L. Chen, Superior oxygen-resistance and intrinsic mechanisms of coherent Pd/Pd_3_Zr@ZrCo structure with excellent cycling durability, Chem. Eng. J. 2024, 479, 147660.

[S2] E.M. Borzone, M.V. Blanco, G.O. Meyer, A. Baruj, Cycling performance and hydriding kinetics of LaNi_5_ and LaNi_4.73_Sn_0.27_ alloys in the presence of CO, Int. J. Hydrogen Energy 2014, 39, 10517-10524.

[S3] G.D. Sandrock, P.D. Goodell, Surface Poisoning of LaNi_5_, FeTi and (Fe,Mn)Ti by O_2_, CO and H_2_O, J. Less-Common Met. 1980, 73, 161-168.
